# The exercise sex gap and the impact of the estrous cycle on exercise performance in mice

**DOI:** 10.1038/s41598-018-29050-0

**Published:** 2018-07-16

**Authors:** Aderbal S. Aguiar, Ana Elisa Speck, Inês M. Amaral, Paula M. Canas, Rodrigo A. Cunha

**Affiliations:** 1 Purines at CNC-Center for Neuroscience and Cell Biology, University of Coimbra, 3004-517 Coimbra, Portugal; 2 Research Group on Biology of Exercise, Department of Health Sciences, UFSC-Federal University of Santa Catarina, Araranguá, SC 88905-120 Brazil; 3 FMUC – Faculty of Medicine, University of Coimbra, 3004-504 Coimbra, Portugal

## Abstract

Exercise physiology is different in males and females. Females are poorly studied due to the complexity of the estrous cycle and this bias has created an exercise sex gap. Here, we evaluated the impact of sexual dimorphism and of the estrous cycle on muscle strength and running power of C57BL/6 mice. Like men, male mice were stronger and more powerful than females. Exercise-induced increase of O_2_ consumption ($\dot{{\bf{V}}}$O_2_) and CO_2_ production ($\dot{{\bf{V}}}$CO_2_) were equal between sexes, indicating that running economy was higher in males. Thermoregulation was also more efficient in males. In females, proestrus increased exercise $\dot{{\bf{V}}}$O_2_ and $\dot{{\bf{V}}}$CO_2_ at low running speeds (30–35% female $\dot{{\bf{V}}}$O_2max_) and estrus worsened thermoregulation. These differences translated into different absolute and relative workloads on the treadmill, even at equal submaximal $\dot{{\bf{V}}}$O_2_ and belt speeds. In summary, our results demonstrate the better muscle strength, running power and economy, and exercise-induced thermoregulation of males compared to females. Proestrus and estrus still undermined the running economy and exercise-induced thermoregulation of females, respectively. These results demonstrate an important exercise sex gap in mice.

## Introduction

The importance of differences between sexes/genders is recognized in biology and medicine. Sex describes biological differences, while gender includes social, cultural and economic aspects^1^. The historical gender differences in motivation/opportunity to practice physical activity (including physical exercise and training) limited the best women exercise/sport performance, a phenomenon known as exercise gender gap in humans^2^. For instance, women are more prone to physical inactivity^3^, a risk factor for many diseases^4,5^. The historical evolution of exercise gender gap in modern Olympic Games (World Record and 10 best performances) also reveals a systematically lower sport performance of females compared to males; nowadays, the differences varies between 10.7% for running and 36.8% for weightlifting^2^. The exercise gender gap is greatest in sports that require running economy, muscle strength, and exercise power^2^. Running economy is the energy demand for a submaximal running speed^6^, higher in men^7,8^ but it is unknown if this sex difference is also present in laboratory animals.

A review of ≈1400 manuscripts involving more than 6 million people revealed an under-representation of women in studies of exercise and sports (35–37%)^9^. However, sex is a major determinant of exercise performance through the impact of anthropometry (height, weight, body fat, and muscle mass), aerobic power and anaerobic threshold, besides genetic and hormonal factors^2–4,10^. The minor representation of females also translates into less knowledge about the biology of exercise in this sex. So far, the main features of sexual dimorphism important for exercise described in rodents are differences in skeletal muscle kinetics and fiber-type composition^10^ and energy metabolism^11,12^. In fact, the biological mechanisms underlying the benefits of exercise were investigated in numerous animal studies in a laboratory setting, with a strong tendency to only use males probably to avoid dealing with the possible influence of the menstrual/estrous cycle^9,13^. Exercise-induced thermoregulation, submaximal and maximal $\dot{{\rm{V}}}$O_2_ and $\dot{{\rm{V}}}$CO_2_, and running economy are gold physiological indexes for exercise, but have never been studied in females at different phases of the estrous cycle.

In humans, the exercise sex gap is greatest in sports that require running economy, strength, and power. Similarly, we investigated the role of sex and estrous cycle in maximum (and submaximal) muscle strength and running power/economy of mice. We also evaluated exercise-induced thermoregulation. This knowledge is essential to advance the knowledge of exercise physiology in female sex. We will demonstrate that the simple extrapolation of male knowledge is not correct.

## Results

### Mouse morphology and the estrous cycle

**Figure 1:**
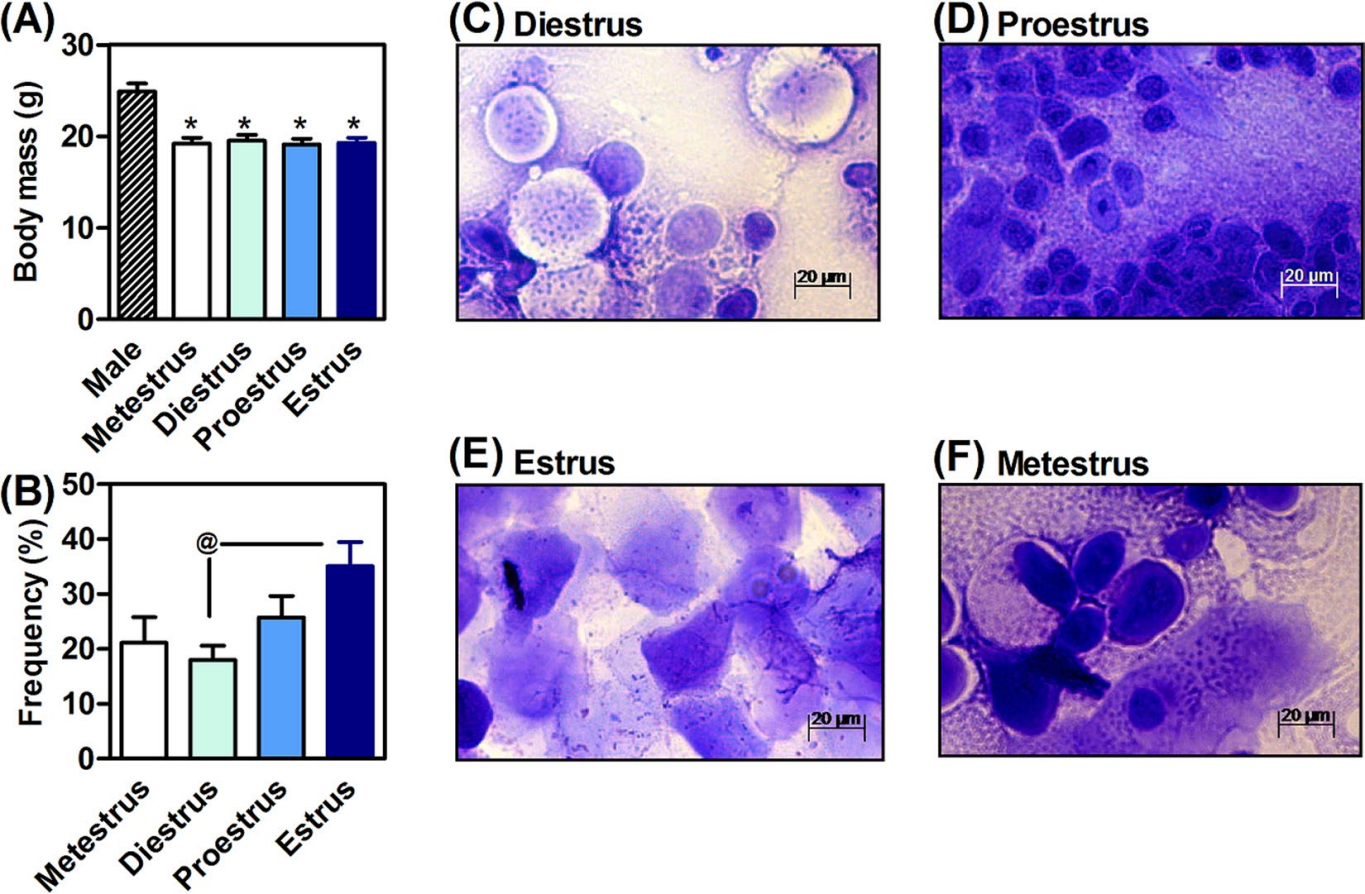
Impact of sexual dimorphism on body mass (**A**) and analysis of the estrous cycle based on a morphological analysis of vaginal smears (**C**–**F**) that revealed that the prominent estrous cycle was estrus (**B**). Values are expressed as mean ± standard error of the mean (SEM). N = 8–10 animals/group. *P < 0.05 *vs*. male (ANOVA, Bonferroni *post hoc* test). ^@^P < 0.05 (Kruskal-Wallis test).

The body mass of males was 22.7 ± 1.1% higher than females (F_4,51_ = 12.6, P < 0.05, Fig. 1A). Rodent tails are important for regulating body temperature^14–16^. The length of the tail of the males (6.5 ± 0.2 cm) was shorter than females (8.1 ± 0.3 cm, t_27_ = 4.5, P < 0.05). These parameters were independent of the estrous cycle, which was devoid of effects on body weight (F_3,43_ = 0.1, P > 0.05; Fig. 1A) and tail length (F_4.51_ = 0.4, P > 0.05; data not shown). The prominent estrous cycle was estrus (H_2_ = 8.1, P < 0.05, Fig. 1B), where vaginal smears were marked by clusters of cornified squamous epithelial cells (Fig. 1E). The vaginal smears also allowed the morphological identification of diestrus, proestrus and metestrus, as exemplified in Fig. 1C,D and F, respectively.

### Male are stronger and more powerful than females

**Figure 2:**
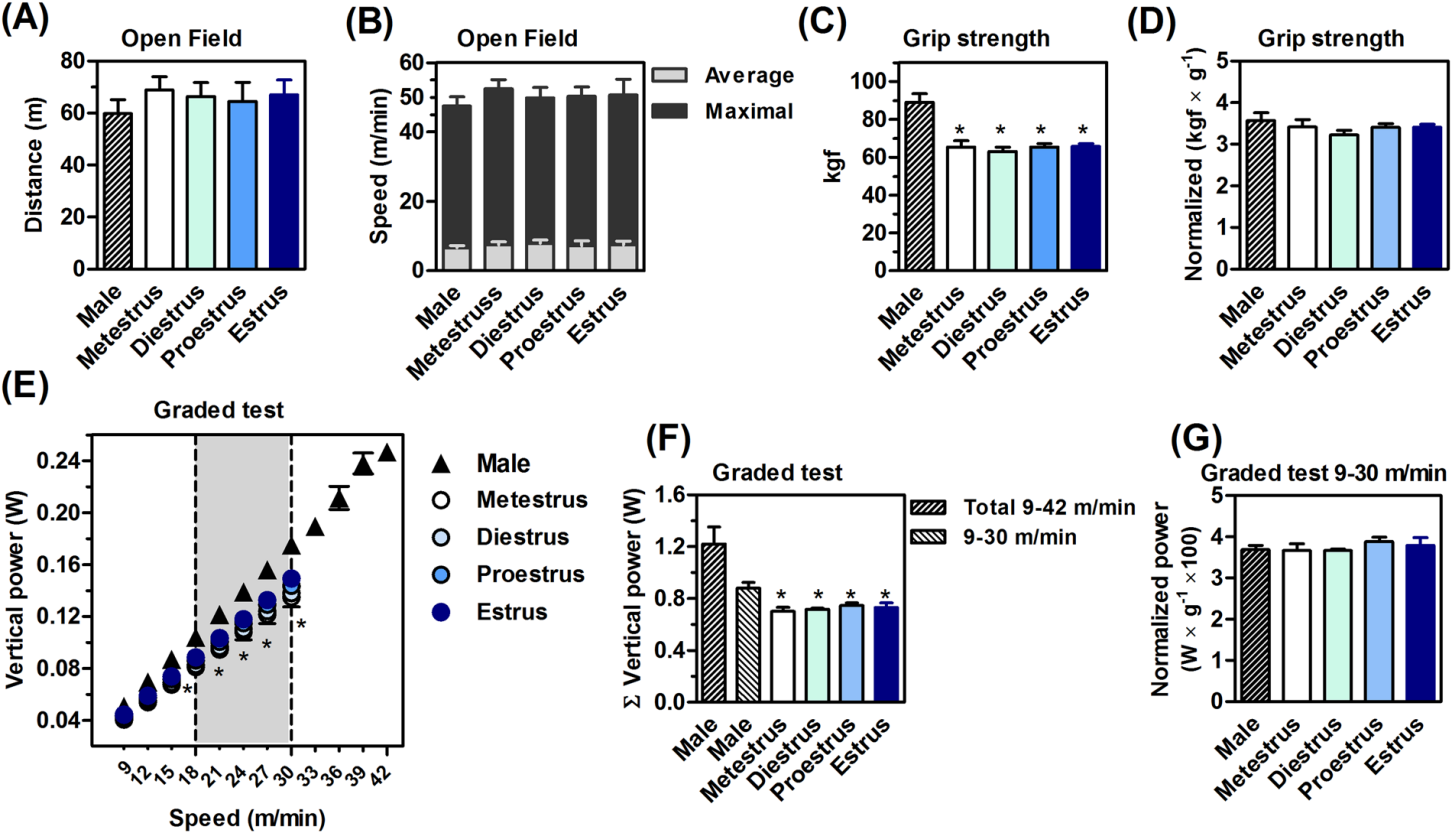
Motor and ergometric data. Sex and estrous cycle did not influence the basal locomotion (**A**) and speed (**B**) of mice. Males were stronger (**C**) and more powerful on the treadmill (**E**,**F**) than females, regardless of the phase of the estrous cycle. The normalization of the performance per body mass dissipated the sexual dimorphism (**D** and **G**). Values are expressed as mean ± standard error of the mean (SEM). N = 8–10 animals/group. *P < 0.05 *vs*. male (ANOVA, Bonferroni *post hoc* test).

Figure 2 shows the basal motor behavior and ergometric performance of male and female mice. The open field test did not reveal significant differences in locomotion (F_4,53_ = 0.39, P > 0.05; Fig. 2A), average (F_4,53_ = 0.38, P > 0.05; Fig. 2B) and maximum speed of males and females, independently of their estrous cycle (F_4,53_ = 0.43, P > 0.05; Fig. 2B).

Absolute exercise performance of females was curtailed in relation to males, being 27.2 ± 1.1% (F_4,32_ = 14.2, P < 0.05; Fig. 2C) and 40.5 ± 0.9% lower (F_4,32_ = 9.9, P < 0.05; Fig. 2F) in the absolute grip strength and treadmill power test, respectively. Moreover, the absolute exercise performance of females was independent of the estrous cycle in the two tests (grip strength F_3,27_ = 0.27, P > 0.05; Fig. 2C) (treadmill power test F_3,27_ = 0.19, P > 0.05; Fig. 2F).

Although the absolute exercise performance of males was higher, the submaximal comparisons indicated a different conclusion. The ergometric test applied progressive running speeds for males and females through serial acceleration (F_21,310_ = 3.2, P < 0.05; Fig. 2E). The treadmill running power in males and females was statistically similar up to 15 m/min (F_21,310_ = 3.2, P > 0.05; Fig. 2E), when the relative intensity was 50 ± 3.7% of the maximum power for females, and 35 ± 3.9%% for males. The lower running power of females appeared at speeds 18 → 30 m/min (F_28,252_ = 18.1, P < 0.05; Fig. 2E, gray area). At 30 m/min, the maximum overload of females (100 ± 5.7%) corresponded to a relative overload of males (71 ± 2.2% of maximum). Males reached maximum overload at speeds 39 → 42 m/min (Fig. 2E).

### The normalization of exercise performance by body mass eliminates sexual dimorphism

We then normalized the exercise performance by the body mass. This transformation eliminated the sex differences for muscle strength (F_4,32_ = 0.78, P > 0.05; Fig. 2D) and running power at speeds 15 → 30 m/min (F_4,32_ = 0.63, P > 0.05; Fig. 2G).

### Males show a better running economy

**Figure 3:**
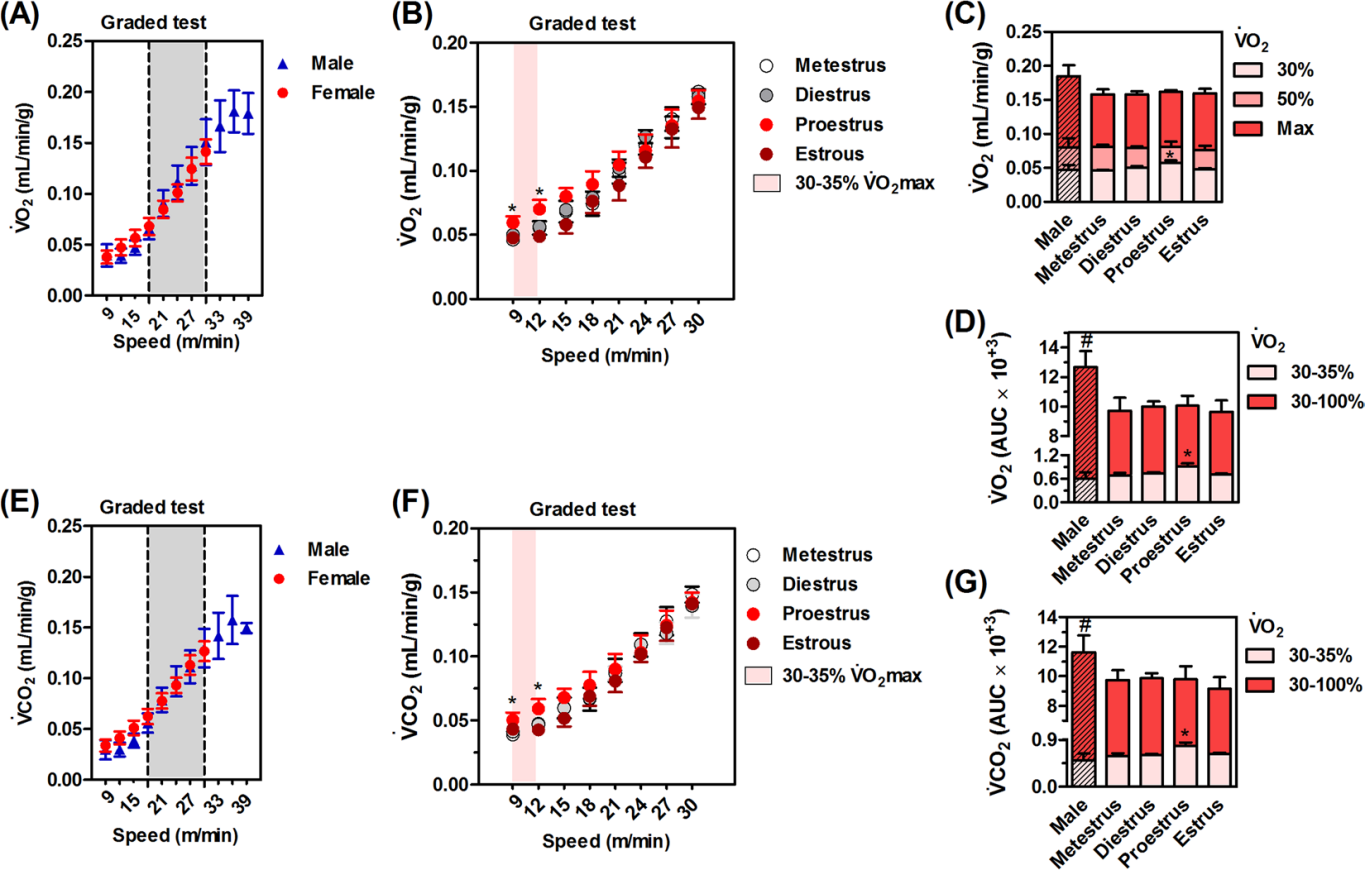
Respiratory gases during an incremental test. Running similarly increased general O_2_ consumption ($\dot{{\rm{V}}}$O_2_, **A**,**B**) and CO_2_ production ($\dot{{\rm{V}}}$CO_2_, **E**,**F**) in males and females at different speeds up to 33 m/min. $\dot{{\rm{V}}}$O_2max_ was similar between the sexes (**C**) total $\dot{{\rm{V}}}$O_2_ (**D**) and $\dot{{\rm{V}}}$CO_2_ (**G**) was only higher in males due to higher running speeds. Proestrus increased submaximal $\dot{{\rm{V}}}$O_2_ (**B** and **D**) and $\dot{{\rm{V}}}$CO_2_ (**G**) at lighter intensities of ergospirometry (30–35% $\dot{{\rm{V}}}$O_2max_). Values are expressed as mean ± standard error of mean (SEM). N = 8–10 animals/group. *P < 0.05 *vs*. male, ^#^P < 0.05 *vs*. females (ANOVA, Bonferroni *post hoc* test).

There were no differences in $\dot{{\rm{V}}}$O_2_ and $\dot{{\rm{V}}}$CO_2_ kinetics between sexes. The progressive running speeds of the ergospirometry increased the $\dot{{\rm{V}}}$O_2_ (F_7,126_ = 2.8, P < 0.05; Fig. 3A) and $\dot{{\rm{V}}}$CO_2_ (F_7,126_ = 2.4, P < 0.05; Fig. 3E) of males and females at all comparative intensities (9 → 30 m/min, Fig. 3A and E). Thus, the higher submaximal running power developed at speeds 18 → 30 m/min, associated to the same submaximal $\dot{{\rm{V}}}$O_2_, showed a better running economy in males compared to females at (Fig. 3A and E, gray area).

Importantly, males ran up to higher speeds (33 → 42 m/min; Fig. 2E), which resulted in a higher $\dot{{\rm{V}}}$O_2_ (F_4,33_ = 2.7, P < 0.05; Fig. 3D) and $\dot{{\rm{V}}}$CO_2_ (F_4,33_ = 2.7, P < 0.05; Fig. 3G), but not $\dot{{\rm{V}}}$O_2max_ in relation to females.

### Proestrus increases $\dot{{\bf{V}}}$O_2_ and $\dot{{\bf{V}}}$CO_2_ during submaximal exercise testing

The submaximal $\dot{{\rm{V}}}$O_2_ (F_18,192_ = 2.5, P < 0.05; Fig. 3B) and $\dot{{\rm{V}}}$CO_2_ (F_18,192_ = 2.3, P < 0.05; Fig. 3C) of females during proestrus were significantly larger at lower exercise intensities (30–35% $\dot{{\rm{V}}}$O_2max_ females. We also detected these differences in total $\dot{{\rm{V}}}$O_2_ (F_3,35_ = 3.8, P < 0.05; Fig. 3D) and $\dot{{\rm{V}}}$CO_2_ (F_3,35_ = 3.2, P < 0.05; Fig. 3G) for females at proestrus during these low exercise intensities (30–35% $\dot{{\rm{V}}}$O_2max_ females). The higher intensities (30–100% $\dot{{\rm{V}}}$O_2max_ females) presented similar kinetics for $\dot{{\rm{V}}}$O_2_ and $\dot{{\rm{V}}}$CO_2_ in the different phases of the estrous cycle.

### Exercise-induced thermoregulation is less effective in estrus females

**Figure 4:**
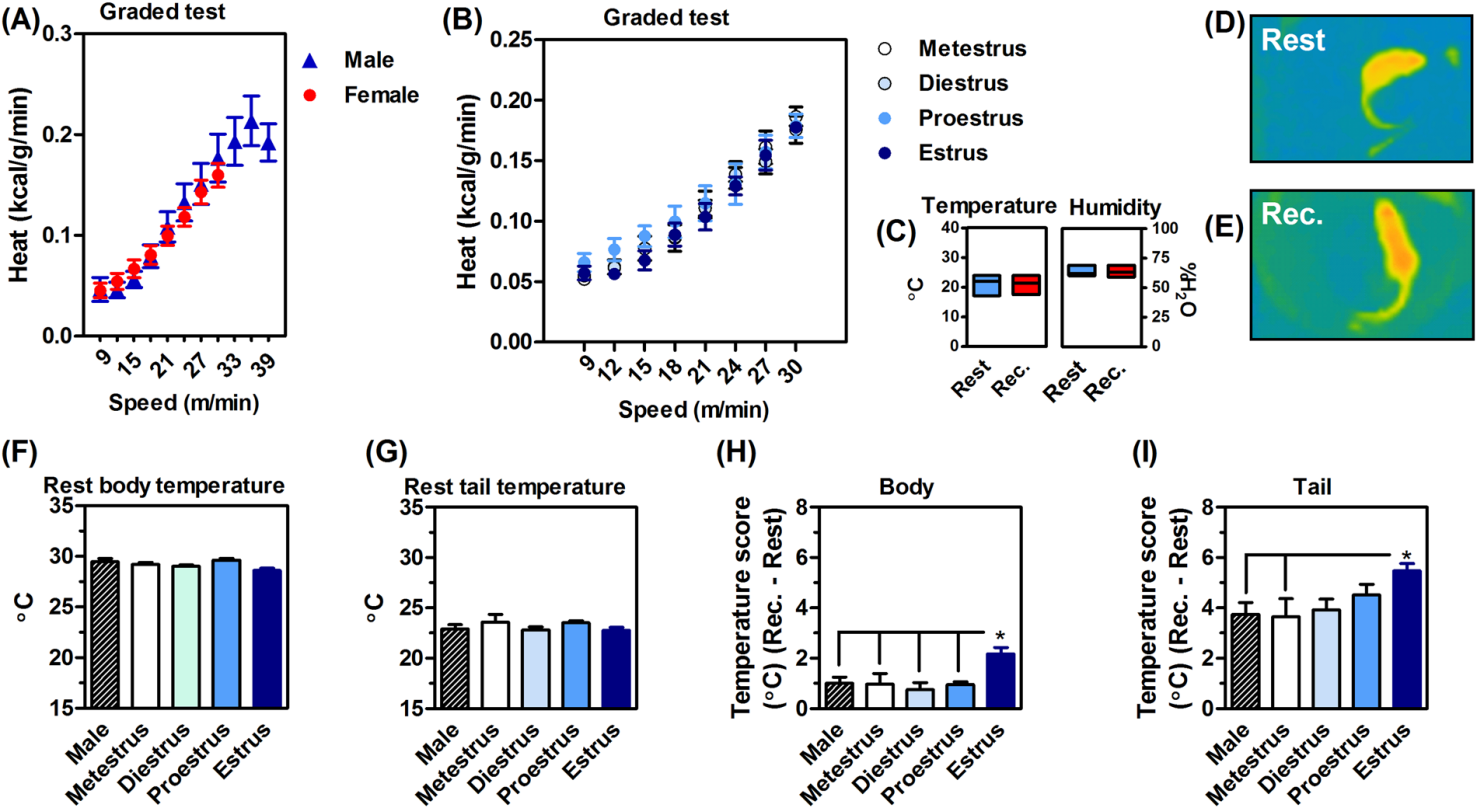
Exercise heat production and dissipation, or thermoregulation. Upon exercise, male and female mice consumed similar calories during the incremental test (**A**) without any evident impact of the estrous cycle. (**B**) Experiments were conducted in a controlled temperature and humidity environment. (**C**) The thermal IR image shows an evident tail heating after the maximum exercise (or recovery time, REC, **E**) in relation to rest. (**D**) The body and tail temperature was not different at rest (**F** and **G**, respectively). Exercise warmed the body of females at estrus (**H**) and the tails of all groups of mice (**I**). Again, female tail heating was larger at estrous (**I**). Values are expressed as mean ± standard error of the mean (SEM). N = 8–10 animals/group. *P < 0.05 (ANOVA, Bonferroni *post hoc* test).

Thermoregulation requires the dissipation of heat produced during exercise. Exercise increased the heat production of males and females (F_7,126_ = 264, P < 0.05; Fig. 4A), without influence of the estrous cycle (F_7,94_ = 0.32, P > 0.05; Fig. 4B). Environment temperature and humidity did not interfere in the thermography results, since they were similar before and after the exercise test session (Fig. 4C). The thermal image (Fig. 4D) shows a female at rest, with the body and tail heated after a maximum exercise test (Fig. 4E).

Resting body and tail infrared temperatures did not differ between sexes or in females at different phases of the estrous cycle (body, F_4,19_ = 0.53, P > 0.05; Fig. 4F) (tail, F_4,19_ = 2.01, P > 0.05; Fig. 4G). The maximum exercise was not enough to heat the body of males and females on metestrus, diestrus and proestrus cycle (F_4,43_ = 3.4, P < 0.05; Fig. 4H). Moreover, all males and females (all cycles) presented significant tail warm up after maximal exercise (F_4,43_ = 2.8, P < 0.05).

The temperature scores (Fig. 4H and I) reinforced the prominent exercise-induced hyperthermia of females at estrus. Estrus female body heating was larger than that of males and females in other cycles (F_4,43_ = 3.3, P < 0.05; Fig. 4H). The tail warming of estrus females was superior to males and females at metestrus after exercise (F_4,43_ = 2.3, P < 0.05; Fig. 4H).

## Discussion

### Sex matters

Sexual dimorphism and the estrous cycle influenced exercise performance and metabolism of mice implying that these factors should be considered in experimental designs and data interpretation involving exercise biology. We showed that males were stronger and more powerful than females at moderate-high intensities of exercise, when evaluating strength and running. Since submaximal and maximum overloads of exercise were different for males and females, but submaximal $\dot{{\rm{V}}}$O_2_ and $\dot{{\rm{V}}}$CO_2_ were similar, this means that the running economy of females was lower than that of males. The estrous cycle did not influence muscle strength, but undermined the running economy and exercise-induced thermoregulation.

### Size matters

The sex-related exercise differences disappeared after normalization of exercise performance by size (body mass). This had already been described for muscle strength^17–19^, but not for running power and economy. However, body size and muscle strength are well-known secondary sexual characteristics, influenced primarily by the anabolic action of the hormone testosterone, a major determinant of sexual dimorphism^20^.

Skeletal muscle mass and strength are lower in females^19,21^. Likewise, normalization of exercise performance by specific muscle mass (rather than body mass) makes sexual dimorphism disappear^19,21^. Male mammals are larger, with larger cross-sectional muscle area^8,10^. Several studies also showed that muscle length (and the length of the long bones) is also higher in male mammals, important for greater tetanic strength of the anterior masseter muscle^8^. Larger levers determine higher torques and muscle strength. Sex is also important for muscle fiber-type composition, especially the myosin IIB gene (fast muscle fiber)^10^. Evidence shows threefold more IIB muscle fibers in the masseter of male mice^8,22^. In addition, testosterone signals hypertrophy in this musculature^20^. Conversely, estrogen decreases muscle contractile force in female mice^23,24^. Thus, muscle strength and running power depends on size and sex: males have large muscles and bones, responsible for great muscle strength; this difference is further amplified by the anabolic effects of testosterone, resulting in larger muscle strength, speed and power.

The testosterone also seems to influence running endurance, but not the running economy. Castration of mouse testicles deplete blood testosterone and impair running wheel endurance (10–30% males with intact gonads)^25^, a model of submaximal physical activity. Testosterone replacement completely reversed this impairment^25^. The antiandrogen Flutamide decreases the treadmill endurance of rats, but does not change $\dot{{\rm{V}}}$O_2max_ and running economy^26^. Here, the exercise-induced submaximal $\dot{{\rm{V}}}$O_2_ and $\dot{{\rm{V}}}$CO_2_ up to $\dot{{\rm{V}}}$O_2max_ were similar between sexes, as previously described^11,12,27^. Only one study demonstrated increased female submaximal $\dot{{\rm{V}}}$O_2_ during treadmill test, which further reinforces the hypothesis of females’ worst running economy^27^. These testosterone evidences support the best physical performance (power and endurance) of running male mice, but not the best running economy.

On the other hand, estrogen seems to influence $\dot{{\rm{V}}}$O_2_ and possibly the running economy of mouse. Similar to our results, submaximal $\dot{{\rm{V}}}$O_2_ was higher in female rats during the estrogen-dominant proestrus at low treadmill speeds 5–12 m/min (6° grade, without acceleration)^17^, which may be considered as a low intensity exercise. We also found these differences at near speeds 9–12 m/min. A possibility is the effect of estradiol in the lung gas-exchange surface area (GSA). $\dot{{\rm{V}}}$O_2_ is directly proportional to GSA^28,29^; which increases during proestrus with high estradiol levels^28,29^. Estradiol also increases lung’s GSA and $\dot{{\rm{V}}}$O_2_ in ovariectomized rats^29^. Our results suggest that estrogen can increase $\dot{{\rm{V}}}$O_2_ during exercise, and worsen the running economy, especially at proestrus.

Exercise-induced hyperthermia is a biological response due to greater muscle activation, mitochondrial uncoupling and proton leak^30^. We now report that sex and the estrous cycle do not modify the calories consumed by exercise, another important variable for running economy; however, our results showed that male thermoregulation was more efficient, since the infrared dissipation of males was more effective. Literature suggests two important points for mouse thermoregulation: body surface area (BSA) and tail dry heat loss. BSA is estimated by the Meeh’s formula (BSA = body weight^0.667^)^25^. The greater body mass of males assists in better heat dissipation during/after exercise. Moreover, tail size seems to be related to thermal stress^14–16,31^, with animals that live in warm environments having longer tails^15,32^. Female tails, even longer, warmed up more during exercise than that of males. The tail length of C57BL/6 female mice was similar to that described in female BALB/c mice^15^. Thus, a longer tail length in female mice is suggestive of a required adaptation to compensate for their lower body mass (and area).

Sanchez-Alavez^33^ demonstrated that body warming during exercise was higher in female mice at estrous. We saw it in the tails. Progesterone promotes heat conservation and higher body temperatures at rest^34,35^. Bilateral ovariectomy eliminated this estrous-associated change^14,33^. We suggest that this may apply to body temperature of running female mice during estrus, characterized by high progesterone levels. Thus, sex seems to be a crucial factor also for the exercise-induced thermoregulation of mice.

Some of our results are similar to those reported in humans, since the physical performance of women is generally lower than in men, in accordance with the exercise gender gap^2,36,37^. The woman’s menstrual cycle is divided into three phases: follicular, ovulation and luteal. The follicular phase can be divided into initial and late, corresponding to metestrus and diestrus, respectively. Ovulation corresponds to proestrus, and the luteal phase to estrus. The woman’s follicular and luteal menstrual cycle does not seem to influence muscle strength, power, and $\dot{{\rm{V}}}$O_2_^1,38–40^. Human studies still allow evaluating rate of perceived effort (RPE), which also does not differ in the different menstrual phases^38,41^. However, the differences we found are close to ovulation, virtually impossible to evaluate in women. We demonstrated that the mouse proestrus (or human ovulation) increased $\dot{{\rm{V}}}$O_2_ and heat production at light exercise.

In summary, our results highlight differences in exercise performance and metabolism between male and female mice. Sex influences size, which appear to be the main factor for mice exercise sex gap. Mouse sexual dimorphism also influenced exercise workload, but not $\dot{{\rm{V}}}$O_2_ and $\dot{{\rm{V}}}$CO_2_, implying a finest running economy in males. Males also presented better thermoregulation after exercise. The estrous cycle played a subtle role in mouse physical performance: proestrus impaired running economy and estrus impaired exercise heat loss. This implies that the impact of the estrous cycle on the performance of females should not be considered a limiting factor for their use in experimental designs. In fact, size is the main factor that should be considered in the construction of experimental designs involving exercising male and female mice. For running, a light-intensity exercise seems similar between the sexes (except proestrus), but the performance of females at moderate-intensity running corresponded to the performance of males at low-moderate intensity; the performance of females at high-intensity running corresponded to the performance of males at moderate-high for males, and male high-intensity running was supra-maximal for females. Failure to consider these differences by measuring only the running speed, as done in most studies, introduces an error to compare performance between sexes. These results are of particular interest to counteract the underrepresentation of females in exercise experimental designs.

## Methods

### Animals

Male and female C57BL/6 mice (10–12 weeks old) were obtained from Charles River (Barcelona, Spain). Mice were housed under controlled environment (12 h light-dark cycle, lights on at 7:00 AM, and room temperature of 21 ± 1 °C) with *ad libitum* access to food and water. Animals were housed and handled according to European Union guidelines and the study was approved by the Ethical Committee of the Center for Neuroscience and Cell Biology (University of Coimbra).

The animals were accustomed to the treadmill for 3 days. The open field or grip strength test was performed on the 4^th^ day in independent groups of animals. Ergospirometry was performed on the 5^th^ day. All tests were carried out between 9:00 and 17:00 hours in a sound-attenuated and temperature controlled observation room under low-intensity light (≈10 lux), where mice had been habituated for at least 1 hour. The apparatuses were cleaned with 10% ethanol between animals. Within the time window of the tests, we did not record any significant impact of the time of day (morning *vs*. afternoon) on the treadmill vertical power, $\dot{{\rm{V}}}$O_2max_ and temperature of the tail at rest in either males or females (data not showed).

### Vaginal cytology

We evaluated the estrous cycle immediately after the behavioral and exercise experiments, through 4–5 consecutive vaginal lavages (with 40–50 μL of distillated H_2_O) then mounted on gelatinized slides (76 × 26 mm). These procedures lasted no more than 3–5 minutes, and there were no major temporal delays between behavioral experiments and fluid collection for vaginal cytology.

The vaginal smear were desiccated at room temperature and covered with 0.1% crystal violet for 1 min, then twice washed with 1 mL H_2_O and desiccated at room temperature. The slides were mounted with Eukitt medium (Sigma-Aldrich) and evaluated under an optical microscope at 1x, 5x and 20x (Zeiss Axio Imager 2). The characterization of the estrous cycle was performed according to literature^20,42^. Females were categorized for initial (metestrus) or late (diestrus) follicular phase, ovulation (proestrus), or luteal phase (estrus)^20,42^.

### Open field

The exploration of an open field (38 × 38 cm) was analyzed for 15 min using the ANY-maze™ video tracking system (Stoelting Co.)^41^.

### Grip strength

The animal was hung with its forepaws to the central position of a 300 g metal grid and the grip strength was determined as the weight pushed (in grams)^41^. The computed result was the average of 3 trials, expressed in kgf.

### Ergospirometry

Mice were accustomed with a single-lane treadmill (Panlab LE8710, Harvard apparatus) for 3 consecutive days (speed 15 cm/s, 10 min, slope 8.7%, 0.2 mA), with 24 h interval between each habituation session.

The ergospirometry test was carried out on 5^th^ day, 48 hours after the last habituation session. The incremental protocol started at 15 cm/s with an increment of 5 cm/s every 2 min, with a constant inclination of 8.7% (5° for the LE8710 model). The exercise test lasted until running exhaustion, defined by the inability of the animal to leave the electrical grid for 5 seconds^43,44^. We estimated the power output for treadmill running based on a standard conversion of the vertical work, body weight and running speed^45,46^. Power is the 1^st^ derivative of work relative to time (run time at each stage).

Oxygen uptake ($\dot{{\rm{V}}}$O_2_) and carbon dioxide production ($\dot{{\rm{V}}}$CO_2_) were estimated during treadmill running in a metabolic chamber^47^ (Gas Analyzer ML206, 23 × 5 × 5 cm, AD Instruments, Harvard) coupled to treadmill. The animals remained in the chamber for 15 min prior to exercise testing. Atmospheric air (≈21% O_2_, ≈0.03% CO_2_) was renewed at a rate 120 mL/min, using the same sampling rate for the LASER oxygen sensor (Oxigraf X2004, resolution 0.01%) and infrared carbon dioxide sensor (Servomex Model 15050, resolution 0.1%). Heat (calories) was estimated according to the equations of Lusk^48^.

### Thermal imaging

An infrared (IR) camera (FLiR C2, emissivity of 0.95, FLiR Systems) placed overtop (25 cm height) of a plastic tube (25 cm diameter) was used to acquire a static dorsal thermal image^49^. IR images were taken immediately before and after exercise tests, namely at rest (Fig. 4D) and recovery (REC, Fig. 4E) periods, respectively. IR images were analyzed with FLiR Tools software (Flir, Boston).

### Tail length

The FLiR C2 camera also captures digital pictures (640 × 480 pixels) that were loaded and calibrated (plastic tube, 25 cm diameter) in the ImageJ software (v1.51j8, NIH, USA) for tail length measurement of live animals (ImageJ software).

### Statistics

Data are presented as mean ± Standard Error of the Mean (SEM). A test for normality was performed by Kolmogorov–Smirnov test. For each test, the experimental unit was an individual animal. The frequency of the estrous cycle was assessed using the Kruskal-Wallis test. The role of sex and estrous cycle in the dependent variables body mass, open field, grip strength and vertical power, $\dot{{\rm{V}}}$O_2_ and $\dot{{\rm{V}}}$CO_2_, and body and tail temperature was evaluated using on-way ANOVA. The repeated measures of ANOVA were performed to evaluate the effect of different treadmill speeds, sex and estrous cycle on the vertical power, $\dot{{\rm{V}}}$O_2_ and $\dot{{\rm{V}}}$CO_2_, and heat. The Bonferroni *post hoc* test was applied for significant F values. The accepted level of significance was p < 0.05. Statistics were performed using Dell Statistica (data analysis software system), version 13.

### Data availability

The datasets generated and analyzed during the current study are available from the corresponding author on reasonable request.
